# P-113 - A TEN-YEAR ANALYSIS OF BLOOD LEAD CONCENTRATIONS (BLC) IN CHILDREN IN SCOTLAND (2015-2024)

**DOI:** 10.1093/pubmed/fdag046.100

**Published:** 2026-07-09

**Authors:** Miss Catriona Wilson, Dr Kate Mark, Dr Carole McRae

**Affiliations:** Public Health Scotland; Public Health Scotland; Public Health Scotland

## Abstract

**Background:**

Lead exposure can have serious consequences for the health of children. There have been significant gains in reduction of lead toxicity in the UK due to legislative practices to reduce lead exposure in the environment. However, cases of lead toxicity persist in Scotland and the rest of the UK. According to laboratory-based surveillance, cases are rising in England. Lead toxicity can be a result, and a driver of, health inequalities, with the most deprived populations more likely to be exposed to lead through poor quality housing.

**Objectives:**

The aim of this study is to improve our understanding of the epidemiology of lead poisoning in children in Scotland.

**Methods:**

All NHS Scotland samples for lead analysis are submitted to the Scottish Trace Elements and Micronutrients Diagnostic and Research Laboratory (STEMDRL). Public Health Scotland (PHS) carried out a retrospective review of all NHS Scotland blood lead results for children under the age of 16 years in the ten-year period from 2015 to 2024.

**Results:**

Findings will be presented for reported blood lead concentrations (BLC) for children aged 0 to 15 years between 2015 and 2024. This will include an analysis of all samples exceeding the child public health intervention threshold of 0.24 μmol/L, presented as rates per million population. Case rates will also be presented by age, sex, geographical location, deprivation, and rurality.

**Conclusions:**

Lead toxicity continues to be a problem for children in Scotland. Intervention case rates have been increasing since 2021. PHS has put in place a laboratory-based surveillance system (Lead in Children Scotland (LiCS)) to capture blood lead results for children in Scotland, and continues to engage with stakeholders to improve prevention, detection and intervention in lead toxicity in children.

